# Massive Intramuscular Haematoma Due to Acquired Factor V Deficiency

**DOI:** 10.1002/jha2.70030

**Published:** 2025-04-14

**Authors:** Lara Budwig, Varvara Bashkirova, Shital Shah, David Maudgil, Dalia Khan, John Willan

**Affiliations:** ^1^ Wexham Park Hospital Frimley Park Hospitals NHS Foundation Trust Wexham UK; ^2^ Oxford Haemophilia and Thrombosis Centre Oxford University Hospitals NHS Foundation Trust Oxford UK

1

A 95‐year‐old retired bus driver presented with a 4‐day history of pain and swelling in the left thigh. There was no history of trauma, infection or medication changes. He had a history of ischaemic stroke, hypertension and diabetes mellitus, he did not drink alcohol in excess, and was previously living independently. His regular medications included clopidogrel, which was stopped on admission. Laboratory tests showed haemoglobin (Hb) of 63 g/L [130–180], prothrombin time (PT) of 68.1s [10.5–14.7] and activated partial thromboplastin time (aPTT) of 209 s [26.4–35.9] with 95% correction on immediate 50/50 mixing studies. Clotting tests had been normal when last tested in 2009. There was no evidence of hepatic dysfunction. Computed tomography of the left thigh (Figure 1 left panel) showed an extensive intramuscular haematoma measuring 28 cm × 10 cm × 10 cm with multiple areas of blushing; there was no evidence of malignancy on full body imaging. Initial management was with fresh frozen plasma (FFP) for a presumed consumptive coagulopathy, along with vitamin K, red cell transfusion and tranexamic acid, however, only minimal improvement in coagulation assays was noted (Figure 1 right panel).

Factor V level (FV), taken after FFP replacement had been initiated, was 1 IU/dL [50–150] with a positive FV inhibitor screen at 0.8 Bethesda Units. Fibrinogen and other clotting factor levels were normal. 1 mg/kg prednisolone was started as first line immunosuppression on day 10.

Despite these interventions, Hb dropped to 55 g/L on day 12 with concern of re‐bleeding into the haematoma. Regular platelet transfusions were initiated (since their alpha‐granules are rich in FV, potentially delivering this factor to the site of primary haemostasis), and 0.4 g/kg doses of intravenous immunoglobulin (IVIg) were given on days 14 and 17. Unfortunately the patient tested positive for SARS‐CoV‐2 on day 15 and clinically deteriorated with renal injury, fluid overload and worsening respiratory infection despite antimicrobial therapy. Plasma exchange was proposed to allow time for the immunosuppression to take effect, but the patient declined this. On day 21 the patient became septic with haemodynamic instability, and passed away the following day.

Acquired FV inhibitors are very rare (estimated incidence of 0.02–0.09 per million persons per year). Triggers include bovine thrombin, antibiotics, infection, malignancies and autoimmune conditions, however up to 30% of cases are idiopathic as in this case. FV levels, inhibitor titre and degree of coagulation profile derangement correlate poorly with likelihood and severity of bleeding, therefore, careful clinical assessment is crucial.

**FIGURE 1:**
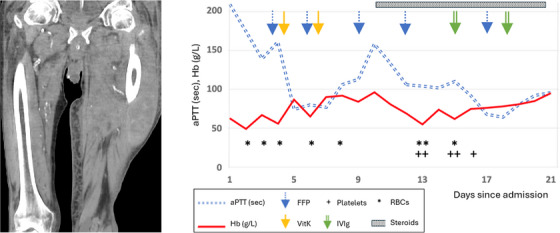
Left: Coronal image of computed tomography scan of patient's thighs, showing an extensive intramuscular haematoma measuring 28 cm × 10 cm × 10 cm with multiple areas of blushing indicating active bleeding. Right: Chart to show changes in aPTT and haemoglobin by time, with therapeutic interventions shown.

## Author Contribution

The paper was drafted by Lara Budwig, Varvara Bashkirova, John Willan. All authors reviewed, edited, and approved the manuscript for publication. The care of the patient was delivered by all authors, with interpretation of radiology images by DM.

## Conflicts of Interest

The authors declare no conflicts of interest.

## Data Availability

The data that support the findings of this study are available from the corresponding author upon reasonable request.
